# Percutaneous vertebroplasty with high- versus low-viscosity bone cement for osteoporotic vertebral compression fractures

**DOI:** 10.1186/s13018-020-01835-y

**Published:** 2020-08-06

**Authors:** Feng Miao, Xiaojun Zeng, Wei Wang, Zhou Zhao

**Affiliations:** Department of Spine Surgery, Renmin Hospital, Hubei University of Medicine, No.39 Middle Chaoyang Road, Shiyan, 442000 Hubei China

**Keywords:** Percutaneous vertebroplasty, Bone cement, Leakage, Viscosity, Osteoporotic vertebral compression fracture

## Abstract

**Objective:**

There is no consensus on the best choice between high- and low-viscosity bone cement for percutaneous vertebroplasty (PVP). This study aimed to compare the clinical and radiological outcomes and leakage between three cements with different viscosities in treating osteoporotic vertebral compression fractures.

**Methods:**

This is a prospective study comparing patients who were treated with PVP under local anesthesia: group A (*n* = 99, 107 vertebrae) with high-viscosity OSTEOPAL V cement, group B (*n* = 79, 100 vertebrae) with low-viscosity OSTEOPAL V cement, and group C (*n* = 88, 102 vertebrae) with low-viscosity Eurofix VTP cement. Postoperative pain severity was evaluated using the visual analog scale. Cement leakage was evaluated using radiography and computed tomography.

**Results:**

There was no significant difference in the incidence of cement leakage between the three groups (group A 20.6%, group B 24.2%, group C 20.6%, *P* = 0.767). All three groups showed significant reduction in postoperative pain scores but did not differ significantly in pain scores at postoperative 2 days (group A 2.01 ± 0.62, group B 2.15 ± 0.33, group C 1.92 ± 0.71, *P* = 0.646). During the 6 months after cement implantation, significantly less reduction in the fractured vertebral body height was noticed in group B and group C than in group A (group A 19.0%, group B 8.1%, group C 7.3%, *P* = 0.009).

**Conclusions:**

Low-viscosity cement has comparable incidence of leakage compared to high-viscosity cement in PVP for osteoporotic vertebral compression fractures. It also can better prevent postoperative loss of fractured vertebral body’s height.

## Introduction

Polymethyl methacrylate (PMMA) bone cement is used in percutaneous vertebroplasty (PVP) for the treatment of osteoporotic vertebral compression fractures. It is injected into the fractured vertebral body to achieve immediate augmentation, relief of the pain, and patient mobility improvement. However, cement leakage into the paravertebral space or blood vessels constitutes a potentially severe complication of PVP and can result in neurological deficit [1] or even paralysis, and pulmonary [2] or heart embolism [3–6], which can be fatal.

Bone cement is prepared by mixing the polymer powder and the monomer liquid of PMMA. The viscosity of the cement paste increases with advancement of polymerization of PMMA until it finally solidifies. The cement is implanted into the fractured vertebra using an injection gun immediately after mixing, when it still has a low viscosity and is easy to aspirate. It has been worried that low-viscosity cement is prone to leak from the vertebra. However, use of high-viscosity cement does not completely prevent the occurrence of leakage [7–11]. In our practice, it was noticed that cement leakage mostly occurs in the late phase of injection, when the cement viscosity is increasing. Increased viscosity requires higher injection pressure and may lead to more leakage. In addition, increased injection pressure causes more intraoperative pain when local anesthesia is used. We speculate that low-viscosity cement may reduce the risk of leakage and have better filling in the vertebral trabeculae and thus prevent postoperative loss of vertebral height.

The present study aimed to compare three cements: high-viscosity OSTEOPAL V, low-viscosity OSTEOPAL V, and low-viscosity Eurofix VTP, in terms of leakage incidence and clinical and radiological outcomes for the treatment of osteoporotic vertebral compression fractures using PVP.

## Materials and methods

### Patients

From March 2015 to February 2018, 226 consecutive patients with vertebral compression fractures who required a PVP were prospectively screened for inclusion in our study. The inclusion criteria were as follows: (1) osteoporotic vertebral compression fracture confirmed by imaging examination, (2) back pain evaluated by visual analog scale above 4 points, (3) bone edema in the fractured vertebra on magnetic resonance imaging (MRI): high signal in T2-weighted images and short tau inversion recovery sequences and low signal in T1-weighted images, (4) age over 50 years; and (5) decreased bone mineral density (T scores < − 1) shown by densitometry.

The exclusion criteria included the following: (1) spinal malignancy, infection, or angioma; (2) spinal cord compression or vertebral canal stenosis more than 30%; (3) neurologic deficits; (4) uncorrectable bleeding disorders; and (5) severe comorbidities of the heart, liver, kidney, or lung. This study was approved by the ethics committee of our hospital.

The patients were divided into three groups: group A (*n* = 99, 107 vertebrae) with high-viscosity OSTEOPAL V cement (Heraeus Medical GmbH, Germany), group B (*n* = 79, 100 vertebrae) with low-viscosity OSTEOPAL V cement, and group C (*n* = 88, 102 vertebrae) with low-viscosity Eurofix VTP cement (Synimed, France). Patient general information was collected from the clinical records. Selection of the type of cement for each patient was at the discretion of the investigators.

### Surgical procedure

The patients were in the prone position. Parecoxib sodium 40 mg and dezocine 5 mg were intravenously administered 30 min before the surgery. Infiltration anesthesia was performed using 0.8–1% lidocaine along the puncture pathway. All surgeries were performed under fluoroscopy by two senior surgeons, Xaojun Zeng and Wei Wang. The destination of the vertebroplasty needle point was the vertebral edema shown by MRI. During the procedure, the needle was stopped and readjusted if severe pain or nerve root irritation occurred. The needle point was advanced to the position medial to the pedicle and near to the base of the spinous process (anteroposterior view) and the anterior one-third borderline of the vertebra (lateral view).

### Cement preparation and implantation

The cement was prepared by mixing the polymer powder and the monomer liquid of PMMA in a dry, clean stainless-steel bowl at an ambient temperature of 22 °C. The high-viscosity OSTEOPAL V cement and the low-viscosity Eurofix VTP cement were prepared exactly according to the manufacturer’s instruction. The low-viscosity OSTEOPAL V cement was prepared by reducing the amount of the polymer powder by 1–2 g. The cement paste was loaded into the injection gun immediately after the preparation process. At around 2 min 5 s after the mixing, the cement was injected into the fractured vertebra under fluoroscopy. The volume of injected cement was recorded.

### Assessment of outcomes

Antiosteoporotic therapy was continued postoperatively, including one Caltrate tablet daily (600 mg of calcium and 125 units of vitamin D3) and alfacalcidol 0.5 μg daily. No analgesics were used postoperatively. The patients were encouraged to ambulate with a wide wrist belt at postoperative 24 h. Cement leakage and filling were evaluated using X-ray and computed tomography (CT) on postoperative day 1. All patients were followed up for at least 6 months with radiography. Pain severity was evaluated preoperatively, intraoperatively, and at postoperative 2 days using the visual analog scale.

**Fig. 1:**
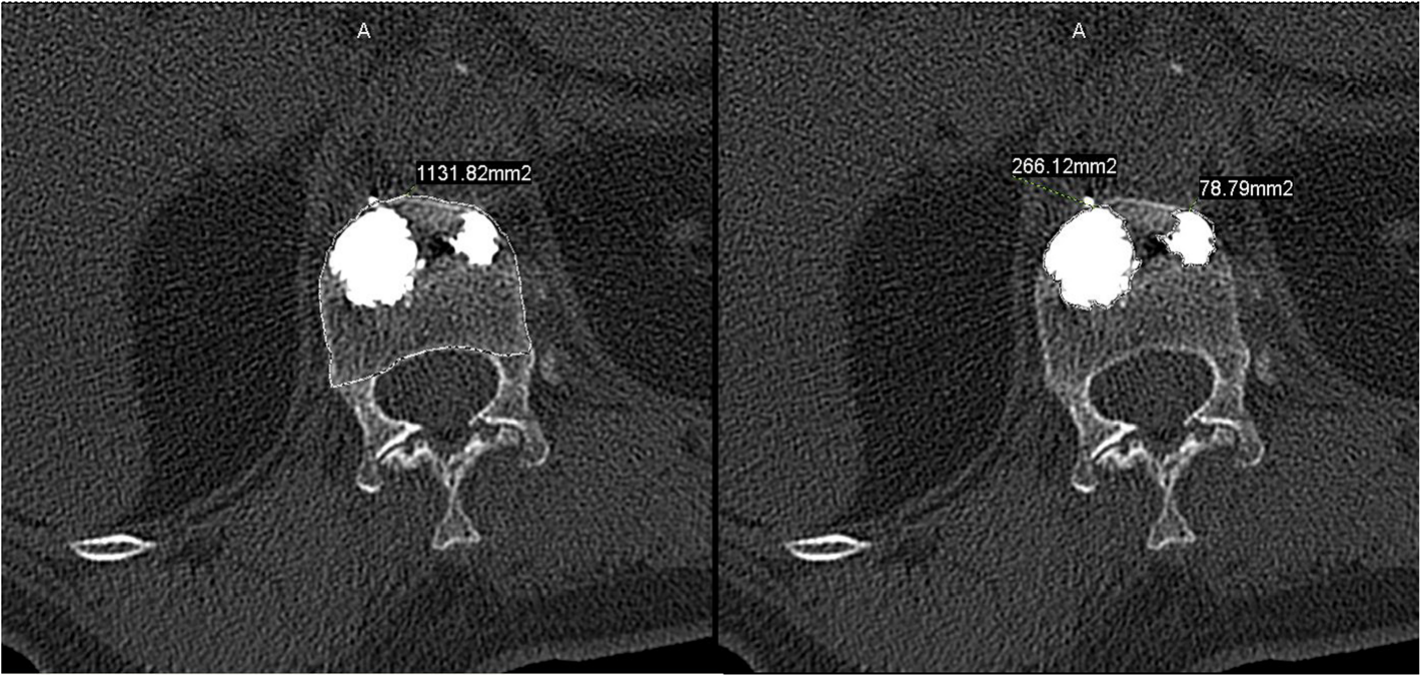
The cement border and the vertebral border in the axial CT images were manually outlined using the “Area measurement” tool in the PACS software, and the areas were automatically calculated

In the axial CT images, the cement border and the vertebral border were manually outlined using the Picture Archiving and Communication System software (Vision series 5.0, AMICAS, Brighton, MA; Fig. 1). Then, the cement diffusion volume and the vertebral volume were automatically calculated by the software by combining the image layers. Cement filling percentage and cement diffusion rate were calculated using the following formulas.

$$ \mathrm{Cement}\ \mathrm{filling}\ \mathrm{percentage}\ \left(\%\right)=\mathrm{cement}\ \mathrm{diffusion}\ \mathrm{volume}\ \left({\mathrm{mm}}^3\right)/\mathrm{vertebral}\ \mathrm{volume}\ \left({\mathrm{mm}}^3\right)\times 100\% $$

$$ \mathrm{Cement}\ \mathrm{diffusion}\ \mathrm{rate}=\mathrm{cement}\ \mathrm{diffusion}\ \mathrm{volume}\ \left({\mathrm{mm}}^3\right)/\mathrm{cement}\ \mathrm{injection}\ \mathrm{volume}\ \left({\mathrm{mm}}^3\right) $$

The anterior vertebral body height was measured in the lateral X-ray images. The Δ percentage of height loss at 6 months was calculated using the following formulas.

$$ \Delta\ \mathrm{percentage}\ \mathrm{of}\ \mathrm{height}\ \mathrm{loss}\ \mathrm{at}\ 6\;\mathrm{months}\ \left(\%\right)=\mathrm{percentage}\ \mathrm{of}\ \mathrm{height}\ \mathrm{loss}\ \mathrm{at}\ 6\;\mathrm{months}\hbox{-} \mathrm{percentage}\ \mathrm{of}\ \mathrm{height}\ \mathrm{loss}\ \mathrm{immediately}\ \mathrm{after}\ \mathrm{the}\ \mathrm{surgery} $$

$$ \mathrm{Percentage}\ \mathrm{of}\ \mathrm{height}\ \mathrm{loss}\ \left(\%\right)=\left(\mathrm{estimated}\ \mathrm{original}\ \mathrm{height}\hbox{-} \mathrm{measured}\ \mathrm{height}\right)/\mathrm{estimated}\ \mathrm{original}\ \mathrm{height}\times 100\% $$

$$ \mathrm{Estimated}\ \mathrm{original}\ \mathrm{height}=\left(\mathrm{height}\ \mathrm{of}\ \mathrm{the}\ \mathrm{superior}\ \mathrm{vertebral}\ \mathrm{body}+\mathrm{height}\ \mathrm{of}\ \mathrm{the}\ \mathrm{inferior}\ \mathrm{vertebral}\ \mathrm{body}\right)/2 $$

### Statistical analysis

All statistical analyses were performed using the SPSS 25.0 (IBM, USA). Continuous data are presented as means and standard deviations and compared using the one-way analysis of variance followed by the Tukey’s post hoc test. Categorical data are presented as percentages and compared using the chi-square test.

## Results

There was no significant difference in the general characteristics between the three groups (Table 1). The three groups showed no significant difference in the incidence of cement leakage (group A 20.6%, group B 24.2%, group C 20.6%, *P* = 0.767; Table 2). No leakage-associated spinal cord injury, nerve root injury, or embolism occurred.

**Table 1:** Patient general characteristics

	Group A (*n* = 99)	Group B (*n* = 79)	Group C (*n* = 88)	*P* value
Cement type	High-viscosity OSTEOPAL V	Low-viscosity OSTEOPAL V	Low-viscosity Eurofix VTP	
Female/male (*n*)	73/26	59/20	65/23	0.989
Age (year)	72.38 ± 9	70.1 ± 7.87	71.5 ± 8.05	0.870
Bone mineral density (T score)	− 2.36 ± 0.67	− 2.89 ± 0.94	− 2.48 ± 0.81	0.659
Number of fractured vertebrae (thoracic/lumbar)	107 (61/46)	100 (56/44)	102 (56/46)	0.954
Total number of vertebral bodies with fissures found in preoperative CT	82	79	80	0.858
Endplate fissure	13	15	16	0.732
Front or lateral wall fissure	71	61	73	0.326
Back wall fissure	17	19	20	0.746

**Table 2:** Cement leakage assessment

	Group A (*n* = 107)	Group B (*n* = 99)	Group C (*n* = 102)	*P* value
Total number of vertebral bodies with cement leakage (%)	22 (20.6)	24 (24.2)	21 (20.6)	0.767
Through endplate	5	6	5	0.892
Through front or lateral wall	9	10	9	0.909
Through back wall	5	4	4	0.959
Though blood vessels	3	4	3	0.863

All three groups showed significant reduction in postoperative pain scores (Table 3). The three groups did not differ significantly in preoperative or postoperative pain scores. However, high-viscosity OSTEOPAL V cement was associated with significantly higher intraoperative pain scores (8.01 ± 1.51) compared to low-viscosity OSTEOPAL V cement (4.01 ± 1.21) and low-viscosity Eurofix VTP cement (4.20 ± 1.16).

**Table 3:** Visual analog scale pain scores

	Group A (*n* = 99)	Group B (*n* = 79)	Group C (*n* = 88)	*P* value
Preoperative	8.31 ± 0.76	8.25 ± 0.57	8.18 ± 0.27	0.872
Intraoperative	8.01 ± 1.51	4.01 ± 1.21	4.20 ± 1.16	0.021
Immediately postoperative	2.11 ± 0.53^a^	2.01 ± 0.69^a^	2.18 ± 0.35^a^	0.735
Postoperative 2 days	2.01 ± 0.62^a^	2.15 ± 0.33^a^	1.92 ± 0.71^a^	0.646
*P* value	0.012	0.011	0.009	

^a^vs preoperative

The volume of injected cement per vertebral body was significantly higher in group B and group C than in group A (Table 4). These two groups also had significantly higher cement filling percentage and cement diffusion rate than group A. At 6 months, there were 12 patients with 12 vertebrae in group A, 10 patients with 11 vertebrae in group B, and 16 patients with 16 vertebrae in group C. The Δ percentage of height loss at 6 months was significantly lower in group B (8.12 ± 0.13) and group C (7.35 ± 0.71) compared to group A (19.01 ± 0.53) (Table 5).

**Table 4:** Cement filling and diffusion

	Group A (*n* = 107)	Group B (*n* = 99)	Group C *(n* = 102)	*P* value
Injected cement volume per vertebral body (ml)				
Through unilateral approach	4.52 ± 2.7	6.92 ± 1.7	6.57 ± 1.9	0.025
Through bilateral approach	7.24 ± 3.1	11.75 ± 2.64	11.41 ± 3.2	0.021
Cement filling percentage (%)	20.78	48.53	49.12	0.017
Cement diffusion rate (%)	4.98	9.55	9.71	0.020

**Table 5:** Further vertebral height loss at postoperative 6 months

	Group A (*n* = 12)	Group B (*n* = 11)	Group C (*n* = 16)	*P* value
Δ percentage of height loss (%)	19.01 ± 0.53	8.12 ± 0.13	7.35 ± 0.71	0.009

## Discussion

Our study found that there was no significant difference in the incidence of leakage and postoperative pain between high-viscosity OSTEOPAL V cement, low-viscosity OSTEOPAL V cement, and low-viscosity Eurofix VTP cement for the treatment of osteoporotic vertebral compression fractures with PVP. The two cements of low-viscosity were associated with significantly less intraoperative pain compared to the high-viscosity OSTEOPAL V cement. They also had significantly higher filling percentage and diffusion rate and significantly less further height loss than the high-viscosity OSTEOPAL V cement.

Viscosity of PMMA can be decreased with extended working time by increasing the liquid-to-powder ratio during the mixing, decreasing the ambient temperature, or chilling the liquid monomer [12]. It has also been reported that increased liquid-to-powder ratio may decrease the mechanical strength of the cement by 24% [13]. However, the clinical impact of this effect is unclear. Two brands of cement were used in our study, OSTEOPAL V and Eurofix VTP. We have tested the low-viscosity OSTEOPAL V cement before our study, which was prepared by reducing the amount of the powder PMMA, and found it had similar mechanical strength to that prepared using the normal liquid-to-powder ratio. Low viscosity of the cement may postpone the solidification and provide more time for cement injection.

The current study found that PVP with either high- or low-viscosity cement significantly reduced postoperative pain with comparable incidence of leakage. Many studies have shown that high-viscosity cement is associated with less leakage-associated complications compared to low-viscosity cement [7, 8, 10, 14–17]. This discrepancy may be caused by the relatively small sample number of our study. In addition, the two low-viscosity cements were associated with significantly less intraoperative pain compared to the high-viscosity cement. We noticed that there was no pain or very mild pain during advancement of the vertebroplasty needle. The patients experienced pain when the cement was injected, especially during the last phase of injection, when the viscosity is increasing and the cement solidifies. Cement implantation increases pressure inside the vertebral body and results in pain during cement injection. Despite the significantly higher volumes of the low-viscosity cements injected, they still resulted in significantly less intraoperative pain compared to the high-viscosity cement without increasing the incidence of leakage. We found that the low-viscosity cement has higher cement filling percentages and higher cement diffusion rates than the high-viscosity cement. This suggests that lower viscosity may ease the spread of the cement through the vertebral trabecular bone, which may reduce bone damage and pain. Further investigation is needed to explain this counterintuitive effect.

In our study, the two low-viscosity cements showed significantly less further height loss than the high-viscosity cement. A previous study also suggested that PVP with low-viscosity cement is superior to high-viscosity cement in restoring the height of the middle vertebra [18]. This may be associated with the significantly higher filling percentage and diffusion rate of the low-viscosity cement and the significantly higher volumes of the low-viscosity cements injected. Lower viscosity may increase the cement infiltration and filling in the vertebral trabeculae, which allows to increase the injection volume. It has been suggested that higher volume of injected cement in the vertebra is associated with better vertebral augmentation and pain relief [19, 20] and less further loss of the vertebral body height [18, 21, 22].

Our study has limitations. First, the cement viscosity was not measured. The relativity of high and low viscosity in our study was determined by altering the liquid-to-powder ratio. Second, only 38 (14.3%) of the patients completed the follow-up at 6 months. This may compromise the reliability of the results of vertebral height loss.

## Conclusions

PVP with low-viscosity cement and high-viscosity had similar incidence of leakage. Low-viscosity cement was associated with significantly less intraoperative pain compared to high-viscosity cement for surgeries under local anesthesia. It can also better prevent the postoperative loss of vertebral height.

## Data Availability

The datasets generated and analyzed during the current study are available from the corresponding author on reasonable request.
